# Non-action Learning: Saving Action-Associated Cost Serves as a Covert Reward

**DOI:** 10.3389/fnbeh.2020.00141

**Published:** 2020-09-04

**Authors:** Sai Tanimoto, Masashi Kondo, Kenji Morita, Eriko Yoshida, Masanori Matsuzaki

**Affiliations:** ^1^Department of Physiology, Graduate School of Medicine, The University of Tokyo, Tokyo, Japan; ^2^Physical and Health Education, Graduate School of Education, The University of Tokyo, Tokyo, Japan; ^3^International Research Center for Neurointelligence (WPI-IRCN), The University of Tokyo Institutes for Advanced Study, Tokyo, Japan; ^4^Brain Functional Dynamics Collaboration Laboratory, RIKEN Center for Brain Science, Saitama, Japan

**Keywords:** reinforcement learning, reward, doing nothing, mouse behavior, decision making

## Abstract

“To do or not to do” is a fundamental decision that has to be made in daily life. Behaviors related to multiple “to do” choice tasks have long been explained by reinforcement learning, and “to do or not to do” tasks such as the go/no-go task have also been recently discussed within the framework of reinforcement learning. In this learning framework, alternative actions and/or the non-action to take are determined by evaluating explicitly given (overt) reward and punishment. However, we assume that there are real life cases in which an action/non-action is repeated, even though there is no obvious reward or punishment, because implicitly given outcomes such as saving physical energy and regret (we refer to this as “covert reward”) can affect the decision-making. In the current task, mice chose to pull a lever or not according to two tone cues assigned with different water reward probabilities (70% and 30% in condition 1, and 30% and 10% in condition 2). As the mice learned, the probability that they would choose to pull the lever decreased (<0.25) in trials with a 30% reward probability cue (30% cue) in condition 1, and in trials with a 10% cue in condition 2, but increased (>0.8) in trials with a 70% cue in condition 1 and a 30% cue in condition 2, even though a non-pull was followed by neither an overt reward nor avoidance of overt punishment in any trial. This behavioral tendency was not well explained by a combination of commonly used Q-learning models, which take only the action choice with an overt reward outcome into account. Instead, we found that the non-action preference of the mice was best explained by Q-learning models, which regarded the non-action as the other choice, and updated non-action values with a covert reward. We propose that “doing nothing” can be actively chosen as an alternative to “doing something,” and that a covert reward could serve as a reinforcer of “doing nothing.”

## Introduction

Animals decide what to do depending on their past experience, and try to act to increase reward and decrease punishment as much as possible (Mazur, 1990). In go/no-go tasks, an action after one cue is rewarded, whereas the same action after another cue is punished (Carandini and Churchland, 2013). After learning the task, animals choose to act in response to the former cue and to not act in response to the latter one. This choice-learning depends on explicitly given (overt) outcomes (Guitart-Masip et al., 2012). In multiple choice tasks, animals choose one of the multiple actions with different outcomes (including reward and/or punishment) in each trial. For example, when an action with a large reward is presented in choice 1 and another action with a small reward is presented simultaneously in choice 2, and these actions have the same cost (e.g., left and right turns), animals choose choice 1. By contrast, when the decision is over choice 2 or another action with a smaller reward (choice 3), animals choose choice 2. Thus, the decision on which action to choose depends on the relative outcome between choices (Wang et al., 2013). Such animal behaviors have been understood in the framework of reinforcement learning, in which the agents learn the policy to take actions (and/or non-action) to maximize some overt reward or avoidance of punishment (Schultz et al., 1997; Sutton and Barto, 1998; Tremblay and Schultz, 2000; Rangel et al., 2008). In this framework, the non-action is considered irrelevant to any overt reward or punishment, and is frequently neglected from the analysis.

However, animals sometimes appear to actively choose non-action, even if the non-action results in no overt reward or avoidance of overt punishment. In such cases, animals may find a positive value (covert reward) in not acting because non-action saves the physical cost of acting and negative feelings such as disappointment and regret that may emerge when action is followed by no reward (Anderson et al., 2003; Niv, 2007; Kühn et al., 2009; Lee et al., 2016; Cheval et al., 2018; Sweis et al., 2018). If this is the case, can learning of the non-action be explained by an increase in the value of the non-action according to such a covert reward? If not, is the non-action chosen because of the reduction in the action value?

To address these issues, we developed a new behavioral paradigm in which head-fixed mice choose to either pull a lever with their right forelimb or to not pull the lever after either of two tone cues with different reward probabilities is presented in each trial (Terada et al., 2018). Although the head-fixed condition was more stressful for the mice than a free-moving condition, we head-fixed them so that two-photon and/or one-photon calcium imaging could be applied during this behavioral paradigm in future experiments to detect the relevant cortical activity.

During learning of this task, the mice decreased their action rate to <0.25 in trials with a cue assigned to a low reward probability when the other trials had a high reward probability, whereas they increased their action rate to >0.8 when the other trials had a much lower reward probability. To explain this behavior, we assumed that the current task was a two-choice task with pull and non-pull choices, and employed several Q-learning models from the theory of reinforcement learning (Schultz et al., 1997; Sutton and Barto, 1998). The behavior was well explained by the models that updated the non-pull value with a covert reward every time the non-pull was chosen. In addition, our models suggest that the subjective goodness of the overt reward depended on the inverse of the total expected outcome included in the task. We propose that the animals learn “not to do,” even if no reward is explicitly presented as the outcome of “not to do,” and no punishment is explicitly given as the outcome of “to do.”

## Materials and Methods

### Animals

All animal experiments were approved by the Institutional Animal Care and Use Committee of the University of Tokyo, Japan. Male C57BL/6 mice (aged 2–3 months at the starting point of the behavioral training; SLC, Hamamatsu, Shizuoka, Japan) were used in the experiments in this study. The mice had not been used for other experiments before this study. All mice were provided with food and water *ad libitum*, and were housed in a 12:12 h light–dark cycle starting at 8 am. All behavioral sessions were conducted during the light period.

### Head-Plate Implantation

Mice were anesthetized by intramuscular injection of ketamine (74 mg/kg) and xylazine (10 mg/kg) before an incision was made in the skin covering the neocortex. After the mice had been anesthetized, atropine (0.5 mg/kg) was injected to reduce bronchial secretion and improve breathing, an eye ointment (Tarivid; 0.3% w/v ofloxacin; Santen Pharmaceutical, Osaka, Osaka, Japan) was applied to prevent eye-drying, and lidocaine jelly was applied to the scalp to reduce pain. Body temperature was maintained at 36–37°C with a heating pad. After the exposed skull was cleaned, a custom head-plate (Tsukasa Giken, Fuji, Shizuoka, Japan) was attached to the skull using dental cement (Fuji lute BC; GC, Bunkyo, Tokyo, Japan; and Bistite II or Estecem II; Tokuyama Dental, Taito, Tokyo, Japan). The surface of the intact skull was coated with dental adhesive resin cement (Super bond; Sun Medical, Moriyama, Shiga, Japan) to prevent drying. An isotonic saline solution with 5% w/v glucose and the anti-inflammatory analgesic carprofen (5 mg/kg, Rimadyl; Zoetis, Parsippany, NJ, United States) was injected once intraperitoneally after the surgery. Mice were allowed to recover for 3–5 days before behavioral training.

### Behavioral Training

After recovery from the head-plate implantation, the mice were water-deprived in their home cages. They received about 1 mL water per session every day, and were sometimes given additional water to maintain their body weight at 80–85% of their initial weight throughout the experiments. The mice were usually trained for five consecutive days per week, and were given a 1.2–1.4 g agar block (Oriental Yeast Co., Ltd., Itabashi, Tokyo, Japan) on days without training. The behavioral apparatus (sound attenuation chamber, head-fixing frame, body holder, sound presentation system, water-supply system, and integrated lever device) was manufactured by O’hara & Co., Ltd. (Nakano, Tokyo, Japan). The lever position was monitored by a magnetic sensor and was continuously recorded at an acquisition rate of 1000 Hz by a NI-DAQ (USB-6001, USB-6221, USB-6229, or PCIe-6361; National Instruments, Austin, TX, United States). The sound control and water delivery were controlled using a program written in LabVIEW (National Instruments).

#### Pre-training

On the first pre-training day, mice were inserted into body chambers and their heads were fixed to the task device for 40 min. Two tones (6 and 10 kHz pure tones, each with a duration of 0.1 s) were alternately presented every three trials. During the first 10–20 trials, a 4 μL drop of water was given from a spout in front of the mice immediately after the tone cues. Over 2–3 days, the mice gradually learned to obtain the water reward by licking the spout after the tone cues. They were then changed to the next task, in which they had to pull the lever more than 1.6 mm for longer than 0.2 s to obtain the reward, instead of just licking the spout. The weight of the lever was fixed at 0.07 N, which was more than twice that used in our previous lever-pull task (0.03 N) (Masamizu et al., 2014). Over 2–5 days, the mice learned to pull the lever for a duration of more than 0.2 s within 1 s after the cue presentation (at 91.9 ± 7.2% of trials after presentation of tone A, and 91.0 ± 10.0% after tone B, in the last session). The mice then started the lever-pull task with different reward probabilities.

#### Lever-Pull Task With Different Reward Probabilities

**FIGURE 1:**
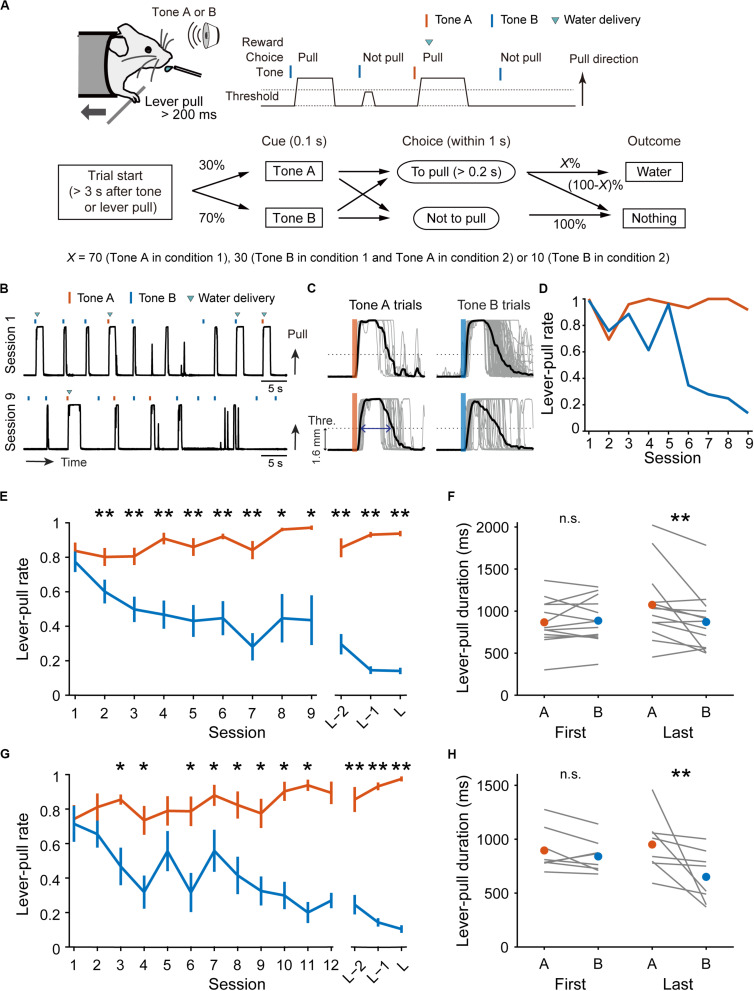
Behavioral task and performance changes over sessions. **(A)** Schematic illustration of the lever-pull task with different reward probabilities. **(B)** Representative lever trajectories of an example mouse in condition 1 in sessions 1 and 9. **(C)** Representative lever trajectories (gray lines) for the same mouse as in **(B)** aligned to the onset of the tone presentation in tone A (left) and B (right) trials with unrewarded successful lever-pulls in sessions 1 (top) and 9 (bottom). A blue arrow indicates the lever-pull duration. **(D)** Representative changes in lever-pull rate for the same mouse as in **(B)** in tone A (red) and B (blue) trials across sessions. **(E)** Changes in mouse-averaged (*n* = 13) lever-pull rate in tone A (red) and B (blue) trials in condition 1. L indicates the last session. *p < 0.05, **p < 0.01, Wilcoxon signed rank test. **(F)** Lever-pull duration in unrewarded trials in the first and last sessions in condition 1. Each line represents an individual mouse, and red and blue dots represent the average of the mice in tone A and B trials, respectively. **p < 0.01, Wilcoxon signed rank test. **(G)** Changes in mouse-averaged (*n* = 8) lever-pull rate in tone A (red) and B (blue) trials in condition 2. *p < 0.05, **p < 0.01, Wilcoxon signed rank test. **(H)** The same as **(F)** in condition 2. **p < 0.01, Wilcoxon signed rank test. See also Supplementary Figure S1.

In the lever-pull task with different reward probabilities (Figure 1A), either of the tone cues used in the pre-training sessions was randomly presented, but tone A was presented in 30% of trials and tone B was presented in 70% of trials. The mice were head-fixed in a way that allowed them to pull the lever within 1 s after the cue presentation, as in the pre-training sessions. The difference from the pre-training sessions was that a different reward probability was assigned to each tone cue. In condition 1, if the mice pulled the lever for longer than 0.2 s, they received a 4 μL drop of reward water at probabilities of 70% and 30% in tone A and B trials, respectively, while in condition 2, the corresponding probabilities were 30% and 10% for tone A and B trials, respectively. If they did not pull the lever, they did not receive the water reward. The next trial started 3–4 s after the last time point at which the lever was returned to the home position (after the lever went below the 1.6 mm threshold), or after the presentation of the previous tone cue when the lever did not exceed the threshold. The presentation probability for tone A was fixed at 30% so that the expected reward per unit of time (if the mice pulled the lever in all trials) was similar between both cues (expected rewards in tone A and B, 0.7 × 0.3 and 0.3 × 0.7 in condition 1, and 0.3 × 0.3 and 0.1 × 0.7 in condition 2).

### Analysis of Behavioral Data

The data were analyzed using MATLAB (MathWorks, Natick, MA, United States). The behaviors of 13 mice were used for condition 1, and eight mice for condition 2. In these mice, the lever-pull choice probability had decreased below 0.25 in tone B trials by training session 20. The latter session of two consecutive sessions in which the mice pulled the lever for more than 80% of tone A trials and less than 25% of tone B trials was set as the last session. No apparent abnormal choice behavior was observed on the day after a break (e.g., on Monday). Therefore, the behavior of the mice was analyzed from the start session to the last session. To omit periods when the motivation of the mice could be considered to be too high or too low within each session, the behavioral data used was taken from the first trial after the mice obtained 30% of the total amount of the reward they got through the session to the last trial before they obtained 70% of the total amount of the reward. The lever-pull rates (the number of successful lever-pull trials divided by the number of presented cues) averaged over the tone A and tone B trials in the early part of each session covering the first 30% of rewarded trials, the middle part of each session covering the 30th–70th percentiles of the rewarded trials, and the late part of each session covering the 70th–100th percentiles of the rewarded trials, were 0.613 ± 0.100, 0.543 ± 0.123, and 0.253 ± 0.087, respectively in condition 1 (*n* = 13 mice), and 0.555 ± 0.101, 0.481 ± 0.097, and 0.367 ± 0.115 in condition 2 (*n* = 8 mice).

Although movement onset latency between the cue presentation and movement onset has frequently been used to estimate attention and reward expectation (Robbins, 2002; Ohmura et al., 2009), it was very similar between the two tone trials in the first session (condition 1, 136.4 ± 28.1 ms vs. 138.4 ± 24.0 ms, *p* = 0.644; condition 2, 174.5 ± 33.7 ms vs. 174.7 ± 28.1 ms, *p* = 0.640, Wilcoxon signed rank test), and was not significantly longer in tone B trials than in tone A trials in the last session (condition 1, 155.1 ± 48.6 ms vs. 202.3 ± 103.4 ms, *p* = 0.094; condition 2, 187.6 ± 53.3 ms vs. 191.8 ± 72.6 ms, *p* = 1.0, Wilcoxon signed rank test). As the movement onset latency was defined as the latency from the cue onset to the first time point that the lever exceeded the threshold, it might be too short to differentiate session-by-session and trial-by-trial variability in latency, even if it existed. Therefore, instead of the movement onset latency, we used the lever-pull duration in unrewarded trials. This was defined as the duration over which the lever trajectories starting within the response window were above the threshold (Figure 1C).

### Reinforcement Learning Models

#### Data Preparation

**FIGURE 2:**
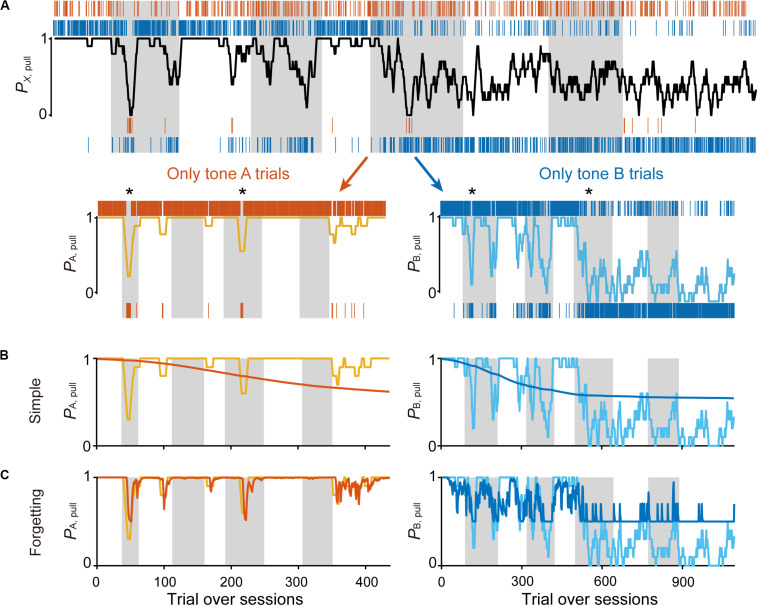
Simple Q-learning model to explain the pull-choice probability. **(A)** Representative time course of the 10-trial moving-average of the pull-choice across nine concatenated sessions in condition 1 (top), and the time course separated into tone A (bottom, left) and tone B (bottom, right) trials. Even sessions are shaded. Asterisks indicate short periods in which the pull-choice probability decreased in both tone A and B trials. **(B,C)** Representative predicted pull-choice probability of the simple **(B)** and forgetting **(C)** models in tone A (left) and B (right) trials. Orange and cyan traces represent the 10-trial moving-average of the actual pull-choice [the same as in **(A)**]. Red and blue traces represent the predicted pull-choice probability.

All behavioral data were summarized as binary data with action (to pull or not), cue type, and reward. The trial sequence in each session was determined by the same criterion as the behavioral analyses. The sequences from a single animal were concatenated through all sessions (Figure 2A, top). The series of data were then separated into two sequences consisting of the same tone cue trials (Figure 2A, bottom), and were used to model the learning process of the mice.

#### Q-Learning Models

We used several extended versions of the Q-learning model (Sutton and Barto, 1998; Barraclough et al., 2004; Ito and Doya, 2009; Guitart-Masip et al., 2012; Akaishi et al., 2014; Skvortsova et al., 2014; Katahira, 2015; Palminteri et al., 2015; Bari et al., 2019), assuming that the current task is a two-choice task with pull and non-pull choices (Guitart-Masip et al., 2012; Swart et al., 2017). First, we built a “simple model” that assumed a value for pulling of the lever *Q*_*X, pull*_(*t*) and a value for non-pulling of the lever *Q*_*X, non–pull*_(*t*) in the *t-*th trial for each tone cue (*X* ∈ {A, B}). *Q*_*X, pull*_(*t*) was updated when the mice pulled the lever as follows:

(1) $$Q_{X,\text{pull}}\,\left(t+1\right)=Q_{X,\text{pull}}\,\left(t\right)+\alpha_{\text{l}}\,\left(R_{X,\text{pull}}\,\left(t\right)-Q_{X,\text{pull}}\,\left(t\right)\right)$$

(2) $$R_{X,\text{pull}}\,\left(t\right)=\kappa_{\text{r}}\,r_{X}\,\left(t\right)$$

where α_l_ is the learning rate, κ_r_ is the subjective goodness of a water reward, and *r*_*X*_(*t*) is 1 when the water reward was delivered, or is otherwise 0 in the *t-*th trial for tone *X*. *Q*_*X, non–pull*_(*t*) was updated when the mice did not pull the lever as follows:

$$Q_{X,\text{non-pull}}\,\left(t+1\right)=Q_{X,\text{non-pull}}\,\left(t\right)$$

(3) $$+\alpha_{\text{l}}\,\left(R_{X,\text{non-pull}}\,\left(t\right)-Q_{X,\text{non-pull}}\,\left(t\right)\right)$$

(4) $$R_{X,\text{non-pull}}\,\left(t\right)=\kappa_{\text{r}}\,r_{X}\,\left(t\right)=0$$

because *r*_*X*_(*t*) was always 0 in non-pull trials. In the “F (forgetting) model” (Barraclough et al., 2004; Ito and Doya, 2009), *Q*_*X, pull*_(*t*) and *Q*_*X, non–pull*_(*t*) were updated in the same manner as in the simple model, but *Q*_*X, pull*_(*t*) in trials without the lever-pull was also updated as follows:

(5) $$Q_{X,\text{pull}}\,\left(t+1\right)=\left(1-\alpha_{\text{f}}\right)\,Q_{X,\text{pull}}\,\left(t\right)$$

where α_f_ is the forgetting rate. In any models without “F” described below, α_f_ was set to zero. *Q*_*X, non–pull*_(*t*) in trials with the lever-pull was updated as follows:

(6) $$Q_{X,\text{non-pull}}\,\left(t+1\right)=\left(1-\alpha_{\text{f}}\right)\,Q_{X,\text{non-pull}}\,\left(t\right)$$

The pull-choice probability for the (*t* + 1)-th trial for tone *X*, *P*_*X, pull*_(*t* + 1), was calculated using the following softmax function:

$$P_{X,\text{pull}}\,\left(t+1\right)=\frac{\exp\left\{Q_{X,\text{pull}}\,\left(t\right)\right\}}{\exp\left\{Q_{X,\text{pull}}\,\left(t\right)\right\}+\exp\left\{Q_{X,\text{non-pull}}\,\left(t\right)\right\}}$$

(7) $$=\frac{1}{1+\exp\left\{-\left(Q_{X,\text{pull}}\,\left(t\right)-Q_{X,\text{non-pull}}\,\left(t\right)\right)\right\}}$$

We set the initial value *Q*_*X, pull*_(1) to be 0.9κ_r_ and *Q*_*X, non–pull*_(1) to be *0* because the mice pulled the lever with the reward probability of 100% in pre-training sessions before the first model-fitted session started. Since *R*_*X, non–pull*_(*t*) was zero in equation (4), *Q*_*X, non–pull*_(*t*) = 0 through the whole sessions in all models, except for the following “saving” and its derivative models. Thus, equation (7) in the “simple” and “F models” equals to

(8) $$P_{X,\text{pull}}\,\left(t+1\right)=\frac{1}{1+\exp\left\{-Q_{X,\text{pull}}\,\left(t\right)\right\}}$$

In the “cost model” and “cost-F model” (Skvortsova et al., 2014), *R*_*X, pull*_(*t*) is calculated as follows:

(9) $$R_{X,\text{pull}}\,\left(t\right)=\kappa_{\text{r}}\,r_{X}\,\left(t\right)-\kappa_{\text{c}}$$

where κ_c_ (≥0) represents the subjective cost accompanying pulling of the lever. *R*_*X, pull*_(*t*) can also be reduced by the aversiveness when the lever-pull is not rewarded. In this case, *R*_*X, pull*_(*t*) can be written as κ_r_*r*_*X*_(*t*)−κ_e_(1−*r*_*X*_(*t*)), where κ_e_ (≥0) represents the subjective emotion evoked by an unrewarded lever-pull and is deformed as follows:

(10) $$R_{X,\text{pull}}\,\left(t\right)=\left(\kappa_{\text{r}}+\kappa_{\text{e}}\right)\,r_{X}\,\left(t\right)-\kappa_{\text{e}}$$

As equations (9) and (10) are mathematically equivalent, we considered only equation (9) as the cost model. *P*_*X, pull*_(*t* + 1) was determined by equation (8).

In the “irregular REL (irREL) model” and “irREL-F model,” we modified the RELATIVE model, which refers to the value of the “context” when updating the *Q*-value (Palminteri et al., 2015; Klein et al., 2017). In our task, we assumed that the “context” was the set of the task, and that in tone A trials, the counterfactual situation was tone B trials, and vice versa. When the mice pulled the lever in the *t*-th trial for tone *X* and the *t*-th trial for tone *X* corresponded to the *t*_AB_-th trial among the total trials including both tone A and B trials, *Q*_*X, pull*_(*t* + 1) was calculated referring to the contextual value *V*(*t*_AB_) calculated with the estimated counterfactual option value *Q*_*Y*__, pull_(*t*_*Y*_) (*Y* (∈ {B, A}) as follows:

$$Q_{X,\text{pull}}\,\left(t+1\right)=Q_{X,\text{pull}}\,\left(t\right)$$

(11) $$+\alpha_{\text{l}}\,\left(R_{X,\text{pull}}\,\left(t\right)-V\,\left(t_{\text{AB}}\right)-Q_{X,\text{pull}}\,\left(t\right)\right)$$

(12) $$V\,\left(t_{\text{AB}}+1\right)=V\,\left(t_{\text{AB}}\right)+\alpha_{\text{v}}\,\left(R\,V\,\left(t_{\text{AB}}\right)-V\,\left(t_{\text{AB}}\right)\right)$$

(13) $$R\,V\,\left(t_{\text{AB}}\right)=\left(R_{X,\text{pull}}\,\left(t\right)+Q_{Y,\text{pull}}\,\left(t_{Y}\right)\right)/2$$

where α_v_ is the update rate of contextual value *V*. *R*_*X, pull*_(*t*) is determined as equation (2). *V* was updated every trial regardless of the tone type, and was used for the update of both *Q*_A, pull_ and *Q*_B, pull_. *Q*_*Y,pull*_(*t*_*Y*_) is the value for the pull for tone *Y* in the tone *Y* trial immediately before the *t*-th trial for tone *X*. When the lever was not pulled in the *t*-th trial for tone *X*, *Q*_*X, pull*_(*t*) was updated according to equation (5). In tone A (or B) trials, *Q*_B,pull_ (or *Q*_A,pull_) was not updated. *P*_*X, pull*_(*t* + 1) was calculated according to equation (8).

In the “saving” and “saving-F” models, *Q*_*X, pull*_(*t*) was updated according to equations (1), (2), and (5), and *Q*_*X, non–pull*_(*t*) was updated when the mice did not pull the lever as in equation (3) and

(14) $$R_{X,\text{non-pull}}\,\left(t\right)=\kappa_{\text{r}}\,r_{X}\,\left(t\right)+\psi$$

where ψ (≥0) is the goodness of the covert reward, which is assumed to be constantly obtained as a result of a non-pull (the saving of the cost accompanying the lever-pull) (Lee et al., 2016; Cheval et al., 2018). When the lever was not pulled, *r*_*X*_(*t*) was zero, so *R*_*X, non–pull*_(*t*) was ψ. *Q*_*X, non–pull*_(*t*) decayed when the mice pulled the lever according to equation (6). The pull-choice probability for the (*t* + 1)-th trial for tone *X*, *P*_*X, pull*_(*t* + 1), was calculated according to equation (7).

In the “O (offset)” model, the point of inflection of the sigmoidal function is offset to the right to let *P*_*X, pull*_(*t* + 1) go to <0.5 when *Q*_*X, pull*_(*t*) = 0, and *P*_*X, pull*_(*t* + 1) is estimated as follows:

(15) $$P_{X,\text{pull}}\,\left(t+1\right)=\frac{1}{1+\exp\left\{-\left(Q_{X,\text{pull}}\,\left(t\right)-\beta_{\text{o}}\,\kappa_{\text{r}}\right)\right\}}$$

where β_o_ (0 < β_o_ < 1) is an offset term of the sigmoidal function (or non-pull bias) and is constant throughout the session. If *Q*_*X, pull*_(*t*) is much smaller than β_o_, *P*_*X, pull*_(*t* + 1) gets close to zero.

In the “I (inertia)” model, we took the history of pull and non-pull choices in the *t*-th trial into account (Akaishi et al., 2014; Katahira, 2018). When the lever was pulled in the *t*_AB_-th trial among the total trials including both tone A and B trials, choice trace *C*(*t*_AB_) was updated as follows:

(16) $$C\,\left(t_{\text{AB}}+1\right)=\left(1-\tau \right)\,C\,\left(t_{\text{AB}}\right)+\tau \,\varphi$$

where *τ* is a decay constant of the choice history (0 ≤ *τ* ≤ 1) and *φ* (>0) is the subjective weight for repeating the same choice. The initial value *C*(1) was set to zero. When the lever was not pulled in the *t*_AB_-th trial,

(17) $$C\,\left(t_{\text{AB}}+1\right)=\left(1-\tau \right)\,C\,\left(t_{\text{AB}}\right)-\tau \,\varphi$$

Therefore, *C* ranged from −*φ* to *φ*. *P*_*X, pull*_(*t* + 1) was calculated as follows:

$$P_{X,\text{pull}}\,\left(t+1\right)$$

(18) $$=\frac{1}{1+\exp\left[-\left\{\left(Q_{X,\text{pull}}\,\left(t\right)-Q_{X,\text{non-pull}}\,\left(t\right)\right)+C\,\left(t_{\text{AB}}\right)\right\}\right]}$$

The parameters used for each model are summarized in Table 1.

**TABLE 1:** Summary of the free parameters used in each Q-learning model.

When the lever was pulled,									
*Q*_*X*,pull_(*t* + 1) = *Q*_*X*,pull_(*t*) + α_l_(κ_r_*r*_*X*_(*t*)−κ_c_−*V*(*t*_AB_)−*Q*_*X*,pull_(*t*))									
*Q*_*X*,non-pull_(*t* + 1) = (1−α_f_)*Q*_*X*,non-pull_(*t*)									
When it was not pulled,									
*Q*_*X*,pull_(*t* + 1) = (1−α_f_)*Q*_*X*,pull_(*t*)									
*Q*_*X*,non-pull_(*t* + 1) = *Q*_*X*,non-pull_(*t*) + α_l_(κ_r_*r*_*X*_(*t*) + ψ−*Q*_*X*,non-pull_(*t*))									
Action probability calculation:									
$P_{X,\text{pull}}\,\left(t+1\right)=\frac{1}{1+\exp\left[-\left\{\left(Q_{X,\text{pull}}\,\left(t\right)-Q_{X,\text{non-pull}}\,\left(t\right)-\beta_{\text{o}}\,\kappa_{\text{r}}\right)+C\,\left(t_{\text{AB}}\right)\right\}\right]}$									
	**α_l_**	**α_f_**	**κ_r_**	**κ_c_**	**ψ**	**α_v_**	**β_o_**	***τ***	***φ***
Simple	var.	0	var.	0	0	0	0	0	0
Forgetting	var.	var.	var.	0	0	0	0	0	0
Cost	var.	0	var.	var.	0	0	0	0	0
irREL	var.	0	var.	0	0	var.	0	0	0
Saving	var.	0	var.	0	var.	0	0	0	0
Cost-F	var.	var.	var.	var.	0	0	0	0	0
irREL-F	var.	var.	var.	0	0	var.	0	0	0
Saving-F	var.	var.	var.	0	var.	0	0	0	0
Cost-F-O	var.	var.	var.	var.	0	0	var.	0	0
irREL-F-O	var.	var.	var.	0	0	var.	var.	0	0
F-O-I	var.	var.	var.	0	0	0	var.	var.	var.
Cost-F-O-I	var.	var.	var.	var.	0	0	var.	var.	var.
irREL-F-O-I	var.	var.	var.	0	0	var.	var.	var.	var.
Saving-F-I	var.	var.	var.	0	var.	0	0	var.	var.
O	var.	0	var.	0	0	0	var.	0	0
I	var.	0	var.	0	0	0	0	var.	var.
F-I	var.	var.	var.	0	0	0	0	var.	var.
Cost-O-I	var.	0	var.	var.	0	0	var.	var.	var.
irREL-O-I	var.	0	var.	0	0	var.	var.	var.	var.
Cost-irREL-F-O-I	var.	var.	var.	var.	0	var.	var.	var.	var.

*var.: variable. When α_*v*_ is zero, *V*(*t*_*AB*_) is zero. When both *τ* and *φ* are zero, *C*(*t*_*AB*_) is zero. See “Materials and Methods” for details.*

#### Model Fitting

Maximum log likelihood estimation was used to fit the parameters used in all models. The likelihood (*L*) was determined using the following formula:

(19) $$L=\prod_{t}z\,\left(t\right)$$

where *z*(*t*) is the likelihood for the *t-*th trial, as follows:

(20) $$\left\{ \begin{array}{cc} z\,\left(t\right)=P\,\left(t\right)\,i\,f\,a\,\left(t\right)=1\; & \\ z\,\left(t\right)=1-P\,\left(t\right)\,i\,f\,a\,\left(t\right)=0\; & \end{array}\right.$$

We took the logarithm of this likelihood and multiplied it by −1 so that we could use the *fmincon* function with appropriate lower and upper bounds for each free parameter in MATLAB.

To compare the models, Akaike’s information criterion (AIC) and Bayesian information criterion (BIC) were calculated using the following formulas (Daw, 2011):

(21) A⁢I⁢C=-2⁢log⁡(L)+2⁢K

(22) B⁢I⁢C=-2⁢log⁡(L)+K⁢log⁢(T⁢n)

where *K* is the number of free parameters to fit, and *Tn* is the number of trials used for fitting.

For visual presentation of time series of the estimated *Q*-values in tone A and B trials in the saving-F model (Figure 9), the values for each animal were normalized by the spline interpolation. The estimated values were up-sampled to the series of 5000 data points by *spline* function, and then averaged across animals in each condition.

#### Model Simulation

To analyze the generative performance of the saving-F and saving-F-I models, we used these models to simulate the lever-pull choice behavior of the mice (Ahn et al., 2008; Palminteri et al., 2015, 2017). For each mouse, the same sequences of tones across sessions were used as in the actual settings, and the fitted values of the free parameters were used as substitutes for the equations above. In each trial, the lever-pull choice (pull or non-pull) was calculated randomly according to the pull-choice probability estimated by equation (7) in the saving-F model, and equation (18) in the saving-F-I model. When the lever was pulled in the simulated *t*-th trial in which it was actually pulled, *r*_*X*_(*t*) was the actual *r*_*X*_(*t*). When the lever was pulled in the simulated *t*-th trial in which it was not actually pulled, *r*_*X*_(*t*) was defined according to the determined probability (condition 1, 70% and 30% in tone A and B trials, respectively; condition 2, 30% and 10% in tone A and B trials, respectively). The initial values of *Q*_*X, pull*_(1), *Q*_*X, non–pull*_(1), and *C*(1) were the same as those for the fitting. The simulation was repeated 1000 times. The lever-pull rate was calculated in the same way as the analysis of the actual behavior and averaged over the 1000 simulations. The goodness of the generative performance was estimated as the proportion of trials in which the simulated pull/non-pull-choice was the same as the actual pull/non-pull-choice.

### Statistical Analysis

Data are presented as mean ± standard deviation unless otherwise indicated. Error bars in the line plots represent the standard error of the mean. The Wilcoxon signed rank test and the Wilcoxon rank-sum test were used for statistical testing in the behavioral analyses. All statistical tests performed were two-tailed.

## Results

### The Mice Chose to Act in Relatively Higher Reward Probability Trials and to Not Act in Relatively Lower Reward Probability Trials

We trained head-fixed mice to perform a lever-pull task with different reward probabilities (Figure 1A). In condition 1, a group of mice (*n* = 13 mice) received a water reward at a probability of 70% after a lever-pull in trials with tone A presentation (tone A trials) and at 30% after a lever-pull in trials with tone B presentation (tone B trials). If they did not pull the lever, they did not receive a water reward. In pre-training, the mice received a water reward every time they pulled the lever after either tone cue was presented (see “Materials and Methods”). As the training days progressed, the lever-pull rate (the session-averaged lever-pull-choice probability) in tone A trials remained high (approximately 0.8), while the lever-pull rate in tone B trials decreased to less than 0.25 (Figures 1B,D,E and Supplementary Figure S1A). In the last session, we also found that the lever-pull duration in unrewarded trials was longer in tone A trials than in tone B trials (Figures 1C,F). These results suggest that as the session progressed, the mice came to expect the reward more strongly, and learned to pull the lever for longer in tone A trials than in tone B trials.

In condition 2, another group of mice (*n* = 8 mice) were trained to perform the lever-pull task with the reward delivered in 30% of lever-pulls in tone A trials and 10% of lever-pulls in tone B trials. As the training progressed, the lever-pull rate in tone A trials in condition 2 increased to >0.8, even though the reward probability was the same (30%) as in the tone B trials in condition 1 (Figure 1G and Supplementary Figure S1B), and the lever-pull rate in tone B trials decreased to <0.25 (Figure 1G and Supplementary Figure S1B). In the last session, the lever-pull duration in unrewarded tone A lever-pull trials was longer than that in tone B trials (Figure 1H). These results indicate that the decision on whether to pull or not does not depend solely on the absolute outcome assigned to each tone.

The number of rewarded lever-pulls per minute was similar between the first session and the last session (condition 1, 2.74 ± 0.92 vs. 2.66 ± 0.53, *p* = 0.735; condition 2, 1.30 ± 0.59 vs. 1.30 ± 0.20, *p* = 0.945, Wilcoxon signed rank test). Although the saving of working time (lever-pull time) could mean more overt (water) reward availability, the similar number of rewards per minute between the first and last sessions suggests that the reason why the mice decreased the pull rate in tone B trials was not because the time saved by skipping the lever-pull in tone B trials increased the overt reward. We postulated that the mice might learn a strategy to not pull in tone B trials to save on the pull-associated cost.

### The Simple Q-Learning Model Does Not Explain the Mouse Choice Behavior Throughout Learning

We then attempted to model these mouse behaviors in the framework of reinforcement learning. In a standard reinforcement learning scheme such as Q-learning, the outcome after action choices is evaluated according to an explicitly given reward (Sutton and Barto, 1998). To apply this to our task, we assumed that the mice chose either of the pull or non-pull choices. When the action value defined as *Q*_action_ and the non-action value defined as *Q*_non*–*action_ are assigned to a sigmoidal function, the action probability, *P*_action_, is written as follows:

(23) $$P_{\text{action}}=\frac{1}{1+\exp\left\{-\left(Q_{\text{action}}-Q_{\mathrm{non}-\mathrm{action}}\right)\right\}}$$

The action has some value because it has a probability of obtaining a reward (that is, *Q*_action_ is positive), whereas the non-action may have no value because it presents no opportunity to obtain the reward (that is, *Q*_non*–*action_ is zero). If so, the action rate should be >0.5 in this equation. We used this simplest Q-learning model (“simple model”) as the starting model to predict the sequence of lever-pull choices concatenated session-to-session for each mouse (Figure 2A; see “Materials and Methods”).

As expected, the predicted pull-choice probability in tone B trials was not below 0.5, and the trial-by-trial fluctuation of the pull-choice probability was poorly predicted by the simple model (Figure 2B). We then introduced the forgetting rate α_f_, which represents decay of the action value when the action is not chosen (Barraclough et al., 2004; Ito and Doya, 2009; Katahira, 2015). In this “forgetting (F) model,” adding α_f_ to the simple model resulted in a better fit to the trial-by-trial fluctuation of pull-choice probability (Figure 2C), although the predicted pull-choice probability in tone B trials was still not below 0.5.

### Assuming That Non-action Saves the Cost of Pulling Explains the Mice’s Behavior

Next, we assumed three improved models with extra parameters to fit the decreased pull-choice probability in tone B trials. In the first “cost model,” we considered that some physical cost accompanied the action (pull). If the expected reward per pull was lower than the physical cost per pull, the pull value would be negative, and as a result, the predicted action choice probability would decrease to <0.5. In the cost model, a constant subjective cost κ_c_ accompanying the lever-pull (since the weight of the lever was constant in the whole session) was introduced into the simple model (Skvortsova et al., 2014).

In the second model, we assumed that the animals updated *Q*_*X, pull*_ according to the relative goodness of the reward outcome in tone *X* trials, comparing it to the expected reward in this task context (Palminteri et al., 2015; Klein et al., 2017). The expected reward in the task context was calculated as the mean of the actual reward in the factual tone trials and the *Q*-value in the counterfactual tone trials (if in tone A trials, consider *Q*_B, pull_, and vice versa). This model is analogous to the “RELATIVE model” (Palminteri et al., 2015), which suggested the relative value compared with the expected reward obtained in the context as the critical decision-making factor, and it well explained the choice action behavior when the different cues were simultaneously presented. Although tones A and B were not simultaneously presented in the current task, we assumed that the mice determined whether to pull according to the difference in the values between the presented and unpresented (factual and counterfactual) tone trials. If *Q*_A, pull_ is much larger than *Q*_B, pull_ in this model, the contextual value (task-environment value) *V* should be larger than the actual reward in tone B trials. Then, *Q*_B, pull_ would be updated to be negative, resulting in *P*_B, pull_ < 0.5 (see “Materials and Methods”). As the different cues are not simultaneously presented, we call this model the “irregular RELATIVE (irREL) model.”

In the third “saving model,” we assumed that the animals might find a positive value (“covert reward”) in the non-action (non-pull) because a non-pull would save the physical cost involved in performing the lever-pull (or allow a rest), and avoid the negative emotion after a lever-pull was not rewarded (Lee et al., 2016; Cheval et al., 2018; Sweis et al., 2018). Thus, in this scenario, if the pull value is smaller than the non-pull value, the predicted pull-choice probability would be <0.5. In the saving model, a constant reflecting the subjective goodness of the cost-saving (as covert reward) ψ accompanying the non-pull was introduced into the simple model, although the mice did not explicitly obtain anything when they did not pull the lever (see “Materials and Methods”).

**FIGURE 3:**
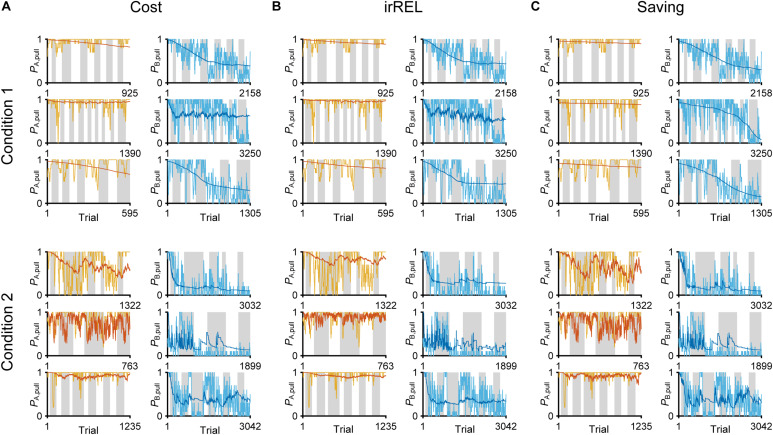
Three Q-learning models to explain the pull-choice probability. Six representative predicted pull-choice probabilities of the models in tone A (left) and B (right) trials in conditions 1 (top three rows) and 2 (bottom three rows). Orange and cyan traces represent the 10-trial moving-average of the actual pull-choice (the same as in Figure 2). Red and blue traces represent the predicted pull-choice probability in cost **(A)**, irREL **(B)**, and saving **(C)** models.

In all three models, the long-term trends in the pull-choice probability fitted well for both tone trials, including the pull-choice probability under 0.5 in tone B trials (Figures 3A–C).

**FIGURE 4:**
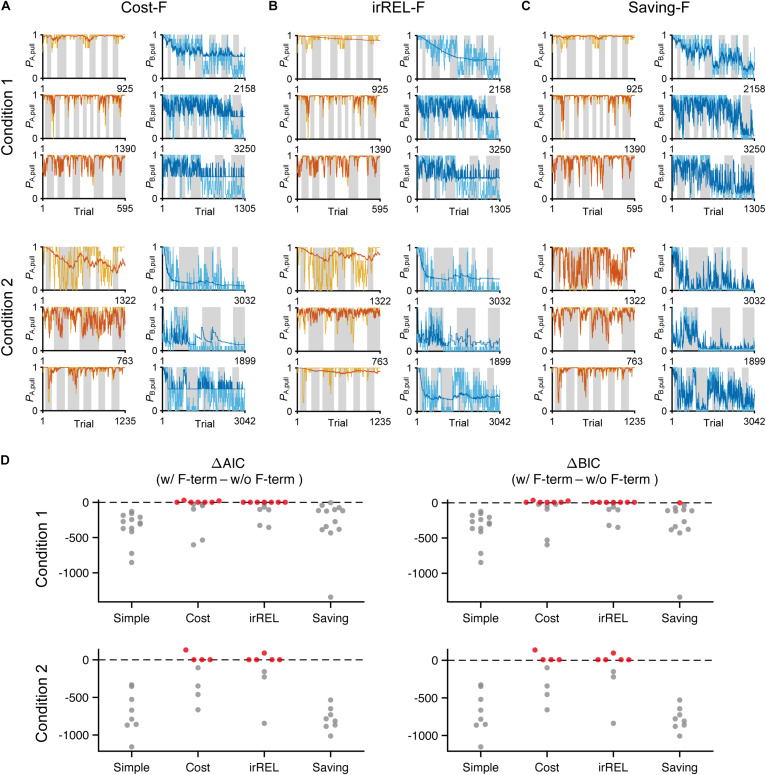
The saving-F model well-explains the pull-choice probability and trial-by-trial fluctuation. **(A–C)** Six representative predicted pull-choice probabilities of the models in tone A (left) and B (right) trials in conditions 1 (top three rows) and 2 (bottom three rows). Orange and cyan traces represent the 10-trial moving-average of the actual pull-choice (the same as in Figure 2). Red and blue traces represent the predicted pull-choice probability in cost-F **(A)**, irREL-F **(B)**, and saving-F **(C)** models. **(D)** Differences in AIC (ΔAIC, left) and BIC (ΔBIC, right) between the models (simple, cost, irREL, or saving) with and without introduction of α_f_ as a free parameter (w/F-term–w/o/F-term). Top, condition 1. Bottom, condition 2. Each dot indicates a single mouse. A negative value indicates that the model prediction with α_f_ was better than that without it. Red dots indicate positive values.

Next, to fit the trial-by-trial variability, we added the forgetting rate α_f_ to the cost, irREL and saving models (cost-F, irREL-F, and saving-F models), and then predicted the pull-choice probability again. In the cost-F and irREL-F models, the fitting of the trial-by-trial variability of the pull-probability in both tone trials appeared to be better than that in the cost and irREL models (Figures 4A,B). However, in approximately half of mice, the pull-choice probability in tone B trials approached 0.5 (Figures 4A,B). This was probably because repeated non-pull behaviors in tone B trials attracted the negative *Q*_B, pull_ to zero by multiplying (1−α_f_) many times. By contrast, in the saving-F models, the prediction of the trial-by-trial fluctuations was better, with the prediction of the lever-pull choice probability of <0.5 being well maintained by all mice in conditions 1 and 2 (Figure 4C and Supplementary Figure S2). Adding α_f_ considerably reduced the AIC and BIC values in the saving model, as well as in the simple model, in almost all mice (Figure 4D). This indicates that α_f_ was an important parameter to explain mouse behavior in the models, especially those in which it did not directly inhibit the prediction of a lever-pull choice probability of <0.5.

**FIGURE 5:**
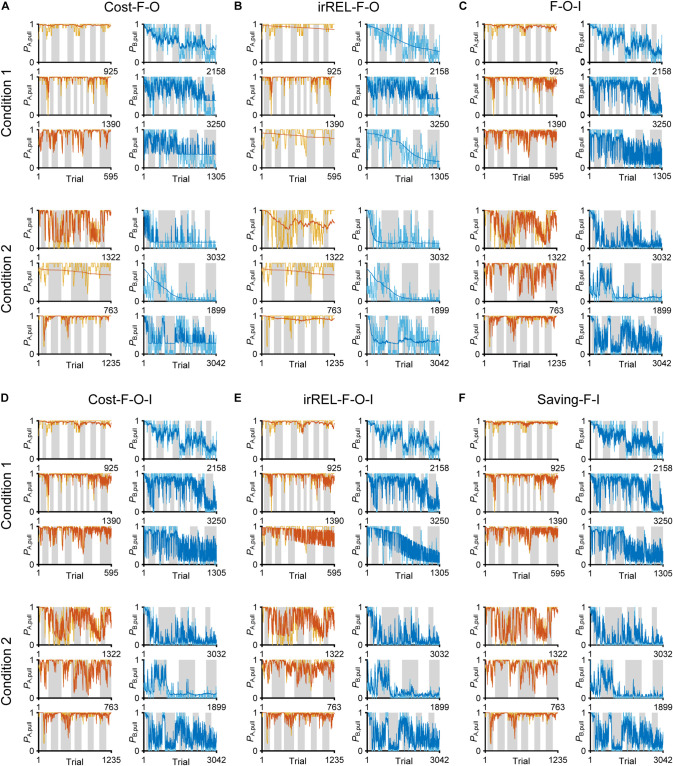
Choice-behaviors predicted by cost-F-O, irREL-F-O, F-O-I, cost-F-O-I, irREL-F-O-I, and saving-F-I models. Six representative predicted pull-choice probabilities of the models in tone A (left) and B (right) trials in conditions 1 (top three rows) and 2 (bottom three rows). Orange and cyan traces represent the 10-trial moving-average of the actual pull-choice (the same as in Figure 2). Red and blue traces represent the predicted pull-choice probability in cost-F-O **(A)**, irREL-F-O **(B)**, F-O-I **(C)**, cost-F-O-I **(D)**, irREL-F-O-I **(E)**, and saving-F-I **(F)** models.

As above, the negative value of *Q*_pull_ in “cost-F” and “irREL-F models” switches the direction of the α_f_ effect (from decreasing to increasing to zero; see “Discussion”). To reduce the pull-choice probability in tone B trials beyond 0.5 in the “+F models,” we also added a parameter to modify *Q*_pull_ after the update; namely, the offset β_o_ was added to the equation for the pull-choice probability (see “Materials and Methods”) to shift the inflection point. If *Q*_pull_ is below β_o_, the pull-choice probability is less than 0.5. The introduction of β_o_ to the cost-F and irREL-F models (cost-F-O and irREL-F-O models, respectively) reduced the pull-choice probability in tone B trials beyond 0.5 (Figures 5A,B). However, the predictions appeared to be worse than those of the saving-F model (Figure 4C).

Over some short periods of time, the choice probability in tone A and B trials appeared to change together (asterisks in Figure 2A). It is reported that animals show persistency in previous choices (inertia) (Akaishi et al., 2014; Bornstein et al., 2017; Bari et al., 2019). To fit the choice behavior better, we further introduced the tendency to repeat the same choice (inertia) into the equation for the choice probability. This inertia derives from the pull and non-pull-choice history, independent of the tone type or reward presence or absence (see “Materials and Methods”) (Akaishi et al., 2014; Katahira, 2018). When this inertia was introduced into the cost-F-O and irREL-F-O models (cost-F-O-I and irREL-F-O-I models, respectively) and their derivatives, the predictions improved (Figures 5C–E and Supplementary Figure S3). Similarly, when the inertia was introduced into the saving-F model (saving-F-I model; see “Materials and Methods”), it also predicted the choice probability well (Figure 5F).

### The Saving-F or Saving-F-I Model Explained the Mouse Choice-Behaviors the Best

**FIGURE 6:**
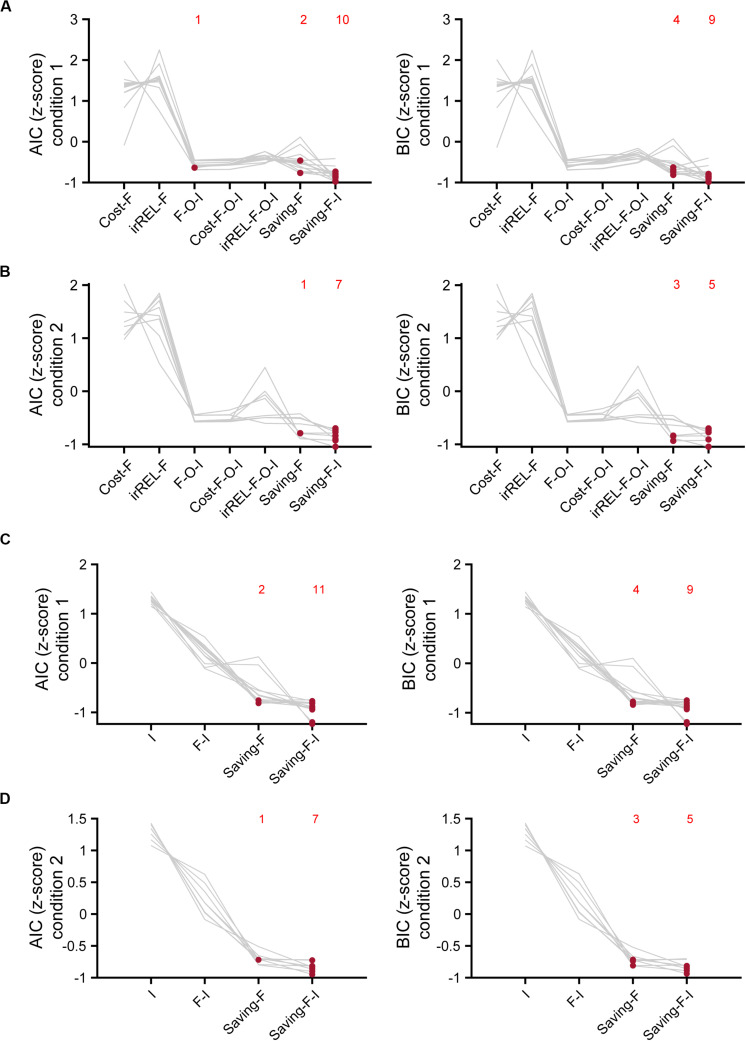
Model comparisons with AIC and BIC. **(A,B)** Z-scored AIC (left) and BIC (right) in cost-F, iREL-F, F-O-I, cost-F-O-I, iREL-F-O-I, saving-F, and saving-F-I models in conditions 1 **(A)** and 2 **(B)**. **(C,D)** Z-scored AIC (left) and BIC (right) in I, F-I, saving-F, and saving-F-I models in conditions 1 **(C)** and 2 **(D)**. Red dots indicate the model with the minimum score for each mouse. Each number indicates the number of the red dots in the corresponding model.

To estimate which model explained the mouse choice-behaviors the best, we used AIC and BIC. The saving-F and saving-F-I models were the best-fitting models in 20 and 21 of the 21 mice in the AIC and BIC comparisons, respectively (Figures 6A,B). When the BIC values were compared, the saving-F model was the best in seven out of 21 mice. In addition, for six out of 14 mice in which the saving-F-I model was the best, the second-best model was the saving-F model. In the seven mice in which the saving-F model was the best, the second-best model was the saving-F-I model in five of the mice. Thus, the second-best model was the saving-F or saving-F-I model in 12 mice. The simple model with the inertia (I model) or forgetting model with the inertia (F-I model) was not better than the saving-F model in any mice except for two in condition 1 (Figures 6C,D). These results suggest that the update of the non-pull value with covert reward, as well as the forgetting rate parameter, was essential to explain the mouse choice-behaviors in this task.

**FIGURE 7:**
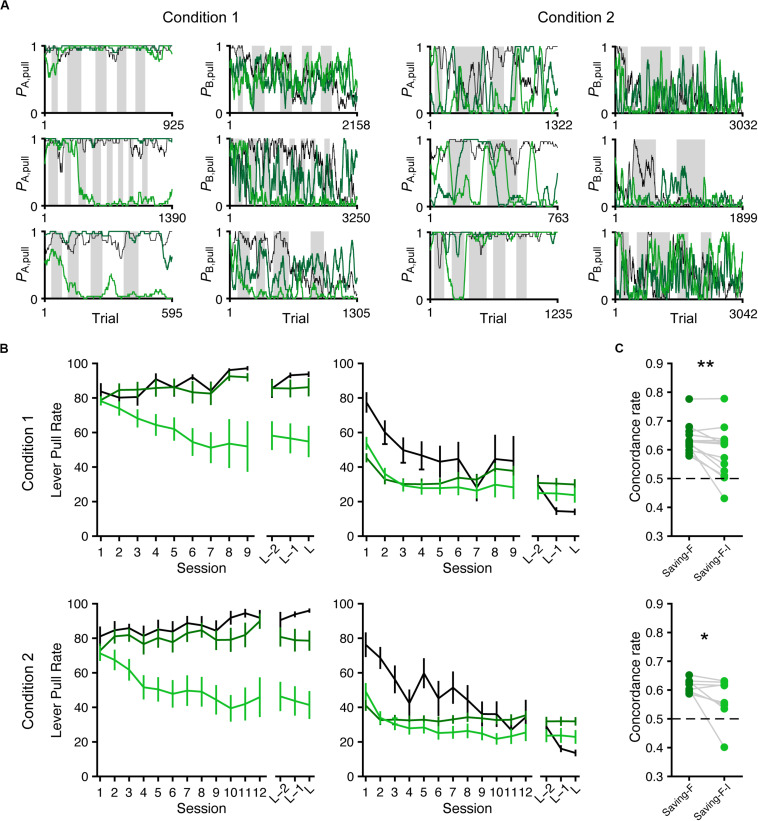
Simulated lever-pull choice behavior with the saving-F and saving-F-I models. **(A)** Six representative simulated pull-choice probabilities with the saving-F (dark green) and saving-F-I (light green) models in tone A (leftmost and second right) and B (second left and rightmost) trials in conditions 1 (leftmost and second left) and 2 (second right and rightmost). Traces represent the 30-trial moving-average of the actual (black) or simulated pull-choice. Even sessions are shaded. **(B)** Mouse-averaged actual (black) and simulated (saving-F model, dark green; saving-F-I model, light green) lever-pull rates in tone A (left) and B (right) trials in conditions 1 (top; *n* = 13 mice) and 2 (bottom; *n* = 8 mice). L indicates the last session. **(C)** The proportion of trials in which the simulated pull/non-pull-choice was the same as the actual pull/non-pull-choice (“concordance rate”) in conditions 1 (top) and 2 (bottom). Each line indicates a single mouse. For each mouse, the concordance rate was calculated for each simulation and averaged over the 1000 simulations. *p = 0.0391, **p = 0.00610.

Next, we conducted model simulation (Ahn et al., 2008; Palminteri et al., 2015, 2017) to examine whether the saving-F and saving-F-I models could generate the across-session choice-behaviors with the lever-pull rate in tone A trials remaining high while the lever-pull rate in tone B trials decreased under both conditions. For each mouse, we used the fitted parameters in the saving-F and saving-F-I models to simulate the lever-pull or non-pull in each trial in the order of the actual tone A and B trials with or without the reward (Figure 7A). The simulation with the saving-F model using the fitted parameters basically reproduced the choice-behaviors, whereas the simulation with the saving-F-I model using the fitted parameters did not generate the behavior, with the lever-pull rate staying high in tone A trials (Figure 7B). The saving-F model simulated the choice-behaviors better than the saving-F-I model (Figure 7C), which suggests that the saving-F model was better as the generative model than the saving-F-I model.

### The Expected Subjective Reward per Action Was Determined by the Inverse of the Expected Overt Reward

**FIGURE 8:**
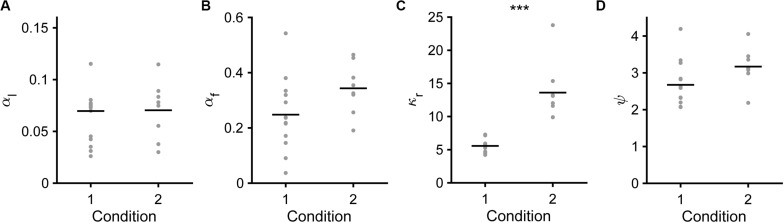
Model parameters in the saving-F model. **(A)** Learning rate (α_l_). Mean ± standard deviation, 0.069 ± 0.037 in condition 1, 0.070 ± 0.028 in condition 2, *p* = 0.587. **(B)** Forgetting rate (α_f_). 0.247 ± 0.131 in condition 1, 0.343 ± 0.092 in condition 2, *p* = 0.0550. **(C)** Subjective goodness of water reward (κ_r_). 5.567 ± 1.101 in condition 1, 13.604 ± 4.469 in condition 2, ***p = 1.92 × 10^–4^. **(D)** Subjective goodness of covert reward (ψ). 2.673 ± 0.626 in condition 1, 3.170 ± 0.521 in condition 2, *p* = 0.0550.

Next, we compared the fitted parameters in the saving-F or saving-F-I model between conditions 1 and 2. In the saving-F model, α_l_, α_f_, and ψ were similar between conditions 1 and 2 (Figures 8A,B,D), which suggests that the mice did not make any changes that would affect these parameters between the conditions with different water reward expectancy per trial under the assumption that the mice pulled the lever in all trials (0.42 = [0.7 × 0.3 + 0.3 × 0.7] vs. 0.16 = [0.3 × 0.3 + 0.1 × 0.7]). By contrast, κ_r_ was more than 2-fold larger in condition 2 than in condition 1 (Figure 8C). In the saving-F-I model, the tendency was also similar, except that α_f_ was larger in condition 2 than in condition 1 (Supplementary Figures S4A–D). The decay and weight of the choice history (*τ* and *φ*) were similar between both conditions (Supplementary Figures S4E,F). The value ranges of α_l_, ψ, and κ_r_ were similar between the saving-F and saving-F-I models, but the values of α_f_ were larger in the saving-F model than in the saving-F-I model. The introduction of choice history might play a role in fitting with the persistency effect from a few preceding choices, without changing the values for the pull and non-pull. By contrast, in the saving-F model, this persistency effect might be substituted by the decay of the value of the non-persistent choice with the larger α_f_. In both models, the ratio of the median κ_r_ in condition 2 to that in condition 1 (2.32 = 13.77/5.93 and 2.75 = 11.89/4.33 in the saving-F and saving-F-I models, respectively) was comparable to the inverse of the expected overt reward per trial in condition 2 divided by that in condition 1 (2.59 = [0.16/0.42]^–1^). These results suggest that the mice recognized the infrequent water delivery as being more valuable than the frequent delivery, while the learning rate and the weight of the choice history did not depend on the combination of the reward probabilities assigned to the tones.

### *Q*-Values for Pull and Non-pull Explained the Different Choice-Behaviors in 30% Reward Probability Cue Trials Between Conditions 1 and 2

**FIGURE 9:**
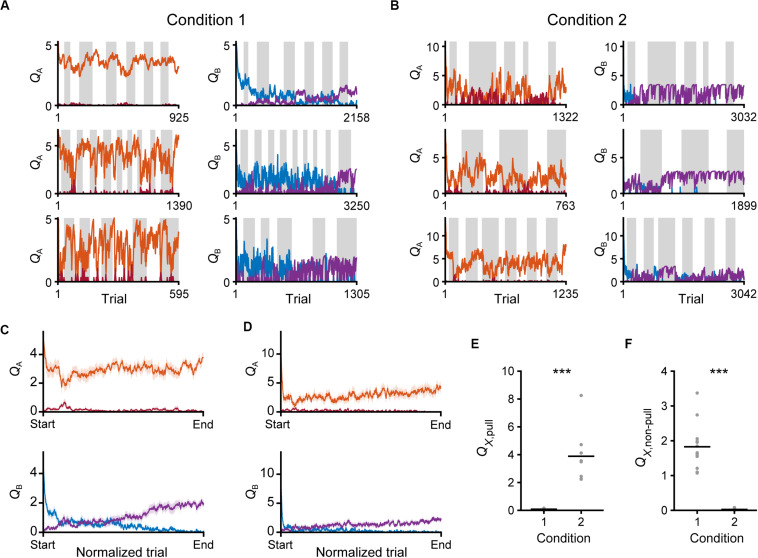
Time courses of *Q*-values in the saving-F model. **(A,B)** Six representative time courses of *Q*_A, pull_ (red) and *Q*_A, non–pull_ (red-purple) (left), and *Q*_B, pull_ (blue) and *Q*_B, non–pull_ (purple) (right), in conditions 1 **(A)** and 2 **(B)**. **(C,D)** The mouse-averaged time courses of *Q*_A, pull_ (red), *Q*_A, non–pull_ (red-purple) (top row), *Q*_B, pull_ (blue) and *Q*_B, non–pull_ (purple) (middle row) in conditions 1 **(C)** and 2 **(D)**. The total trial number was normalized across animals. Shading indicates ± SEM. **(E)** Pull values in tone *X* trials in the last session. *X* is B in condition 1 and A in condition 2. ***p = 1.92 × 10^–4^. **(F)** Non-pull values in tone *X* trials in the last session. *X* is B in condition 1 and A in condition 2. ***p = 1.92 × 10^–4^. Gray dots represent individual mice, and black bars represent the mean.

We evaluated the changes in *Q*-values in the saving-F model. Throughout the training sessions, *Q*_A, pull_ still remained high, while *Q*_B, pull_ gradually decreased in both conditions 1 and 2 (Figures 9A–D). By contrast, *Q*_A, non–pull_ remained low and *Q*_B, non–pull_ gradually increased (Figures 9A–D). Thus, both the values for the pull and non-pull appeared to be acquired through learning. Although the reward probability in tone A trials in condition 2 was lower than that in condition 1, the time course of *Q*_A, pull_ was similar between conditions 1 and 2 (Figures 9C,D). This was probably because the larger κ_r_ in condition 2 increased *Q*_A, pull_ per rewarded lever-pull trial more than κ_r_ in condition 1 did. The time course of the gradual increase in *Q*_B,__non*–*__pull_ was also similar between conditions 1 and 2 (Figures 9C,D). This was probably because the increase in *Q*_B, non–pull_ by ψ per non-pull was similar between these conditions. The model suggests that these *Q*-value changes were the basis of the similarity in the time course of the lever-pull rate between the same tone trials in conditions 1 and 2 (Figures 1E,G).

Finally, we compared the *Q*-values of the trials with a reward probability of 30% in the last session between conditions 1 and 2. Although *Q*_B, pull_ in condition 1 and *Q*_A, pull_ in condition 2 were the values for the actions in trials with the same reward probability, only the former was near to zero (Figure 9E). In concert with this, *Q*_A, non–pull_ in condition 2, but not *Q*_B, non–pull_ in condition 1, was near to zero (Figure 9F). As expected from the large difference in κ_r_ between conditions 1 and 2, the expected subjective reward per action (0.3 × κ_r_) was smaller than the median ψ in condition 1 (0.3 × 5.97 < 2.6), and vice versa in condition 2 (0.3 × 13.77 > 3.36). These relationships were the same in the saving-F-I model (Supplementary Figures S4G,H). These results suggest that the non-action could be more valuable than the action in trials with a reward probability of 30% in condition 1, but less valuable in those trials in condition 2. Because of the effect of α_f_, the values of the unchosen lever-pull (*Q*_B, pull_) in condition 1 and the unchosen non-lever-pull (*Q*_A, non–pull_) in condition 2 became close to zero in the last session. Together with the previous section, the same reward probability-assigned trials induced different choice behavior, which could be determined by the inverse of the expected overt reward through the task.

## Discussion

In this study, we developed a new behavioral paradigm to let mice choose to pull or not pull a lever according to tone cues with different reward probabilities. We found that they came to not pull in relatively lower-reward-expected trials, although the predicted behavior according to an explicitly given reward-maximization policy would be to pull the lever in all the trials unless skipping tone B trials would result in an increase in the total rewards per unit time. To explain the mouse choice-behaviors, we constructed several Q-learning models, in which the pull value was updated by the overt reward, the non-pull value was updated by the covert reward, the value of the unchosen option decayed, the pull value was reduced according to the pull cost, the relative pull value was based on the tone context, and the pull offset (or non-pull bias) and/or the inertia of the choice history was included. We found that the best models were saving-F or saving-F-I models that updated the pull value with the overt water reward, updated the non-pull value with the covert reward, and the value of the unchosen choice decayed. To the best of our knowledge, the current study is the first attempt to indicate that a covert reward might be engaged in non-action learning using a reinforcement learning framework.

In the saving-F and saving-F-I models, when the animal repeatedly chose the non-pull, *Q*_*X, non–pull*_ increased while *Q*_*X, pull*_ decreased to zero. Thus, the non-pull-choice was maintained, which was different to the other models. Even when the action offset and/or the choice inertia were added to the simple, F, cost, and irREL models and their derivatives, the predictions were worse than that of the saving-F-I model. The prediction by the saving-F model was better than those by the I model and F-I model. Thus, we conclude that the active learning of non-action with the covert reward contributed to decision-making. The advantage of the saving-F models may be that the animal can increase its preference to either the pull or non-pull direction in each trial, allowing the choice preference to converge to the final one faster than it can with the models without the non-pull value updated by covert reward. The effectiveness of the choice history on the prediction in the saving-F-I model might reflect the fact that the mice tended to be persistent in pull or non-pull choices for a few trials. However, the saving-F model was better than the saving-F-I model as the generative model to describe the behavioral effect. In the simulation with the saving-F-I model, the averaged lever-pull rate in tone A trials was around 0.5 in condition 1 and <0.5 in condition 2. Tone B was more presented than tone A, and the mice chose not to pull in tone B trials as the sessions progressed. This might result in that the inertia of non-pull-choice in tone B trials inhibited maintenance of the high lever-pull rate in tone A trials in the simulation, and/or the saving-F-I model was overfitted. The role of inertia needs further validation in future studies.

Non-action has long been discussed in the context of response inhibition in go/no-go tasks and stop-signal tasks (Tremblay and Schultz, 2000; Kühn and Brass, 2009; Schel et al., 2014), and several models assumed that subjects updated the value for non-action on the basis of explicitly given reward or punishment (Guitart-Masip et al., 2012; Collins and Frank, 2014; Kato and Morita, 2016; Swart et al., 2017). The use of a covert reward instead of an overt reward to learn a non-action has not yet been fully discussed in standard reinforcement learning. However, in the real world, we appear to actively choose non-action to save on costs such as fatigue accompanying the action, even if the non-action produces nothing (Lee et al., 2016). A human study proposed that avoidance of an overt aversive outcome can in itself be a reward for learning avoidance of that action (Kim et al., 2006). Our results suggest that our animals could actively choose the non-action by evaluating the non-action in the form of the covert reward. Our study sheds light on the favorable aspect of non-action through preventing an aversive cost (or energy) inevitably related with the action, and proposes that non-action could be reinforced by itself.

In the saving-F and saving-F-I models, there were two subjective parameters for the value update, the subjective covert reward (ψ) and the subjective goodness of the overt reward (κ_r_). The physical cost included in ψ would be related to the lever weight, lever-pull length, and lever-pull duration. These might be equivalent to repeated pressing of a lever (Walton et al., 2006; Randall et al., 2012; Sommer et al., 2014) and climbing a wall or barrier (Walton et al., 2002; Floresco and Ghods-Sharifi, 2006; Yohn et al., 2015) in previous effortful decision-making tasks. To validate ψ, it might be useful to examine the correlation between the value of ψ and quantifications of the lever weight, lever-pull length, and lever-pull duration. In contrast to ψ, which was similar between conditions 1 and 2, κ_r_ was ∼2.5-fold larger in condition 2 than in condition 1, which is in inverse proportion to the net reward expectancy when the mice pulled in all trials regardless of tone types. This suggests that the mice recognized the infrequent water delivery as being more valuable than the frequent delivery. This may be regarded as being analogous to a puddle found in the desert. It is necessary to examine the relationship between κ_r_ and the net overt reward using other combinations of reward probabilities.

Comparison between the neuronal activity and *Q*_*X, non–pull*_ would allow us to clarify the neuronal activity relevant to the non-action learning with the covert reward. The striatum and orbitofrontal cortex may be the candidate areas for this neuronal activity because these areas are strongly related to the value update, and activation of the orbitofrontal cortex is related to response inhibition (Tremblay and Schultz, 2000; Yoshida et al., 2018; Jahfari et al., 2019). As the present task is for head-fixed mice, both two-photon calcium imaging with a high spatial resolution (Horton et al., 2013; Masamizu et al., 2014; Kondo et al., 2017; Tanaka et al., 2018) and wide-field calcium imaging (Ghanbari et al., 2019) could be applied during task performance, as well as electrical recording. It would be possible to examine the information flow from the auditory cortex to the forelimb motor cortex through the striatal and orbitofrontal cortical areas. In addition, examination of which brain areas represent ψ and κ_r_ is a task for future studies. If the relationship between the neural activity and these parameters is weak, it would be better to consider the model parameters as variable (Ito and Doya, 2009), because the parameters used in the current models could change during learning. We hope that the saving model will be verified and modified in many tasks including a “not to do” choice, and that it will be helpful for understanding the decision-making process of “not to do” across species. The covert reward concept might also be applicable to understanding the mechanism of social withdrawal and its care, as it is said that socially withdrawn people tend to choose not to go out (Rubin et al., 2009; Li and Wong, 2015).

## Data Availability Statement

The raw data supporting the conclusions of this article will be made available by the authors, without undue reservation.

## Ethics Statement

The animal study was reviewed and approved by the Institutional Animal Care and Use Committee of the University of Tokyo.

## Author Contributions

ST, MK, and MM designed the experiments. ST conducted all the experiments. MK conducted the preliminary experiment and improved the experimental devices and the software. ST and MK constructed the models with KM and EY. ST, MK, and MM wrote the manuscript, with comments from KM and EY. All authors contributed to the article and approved the submitted version.

## Conflict of Interest

The authors declare that the research was conducted in the absence of any commercial or financial relationships that could be construed as a potential conflict of interest.
