# Supplementary material for: Non-action Learning: Saving Action-Associated Cost Serves as a Covert Reward
Source: Front Behav Neurosci. 2020 Sep 4;14:141. doi: 10.3389/fnbeh.2020.00141 (PMC7498735; doi:10.3389/fnbeh.2020.00141)
Supplement: Supplementary file 1 [file Presentation_1.pdf]

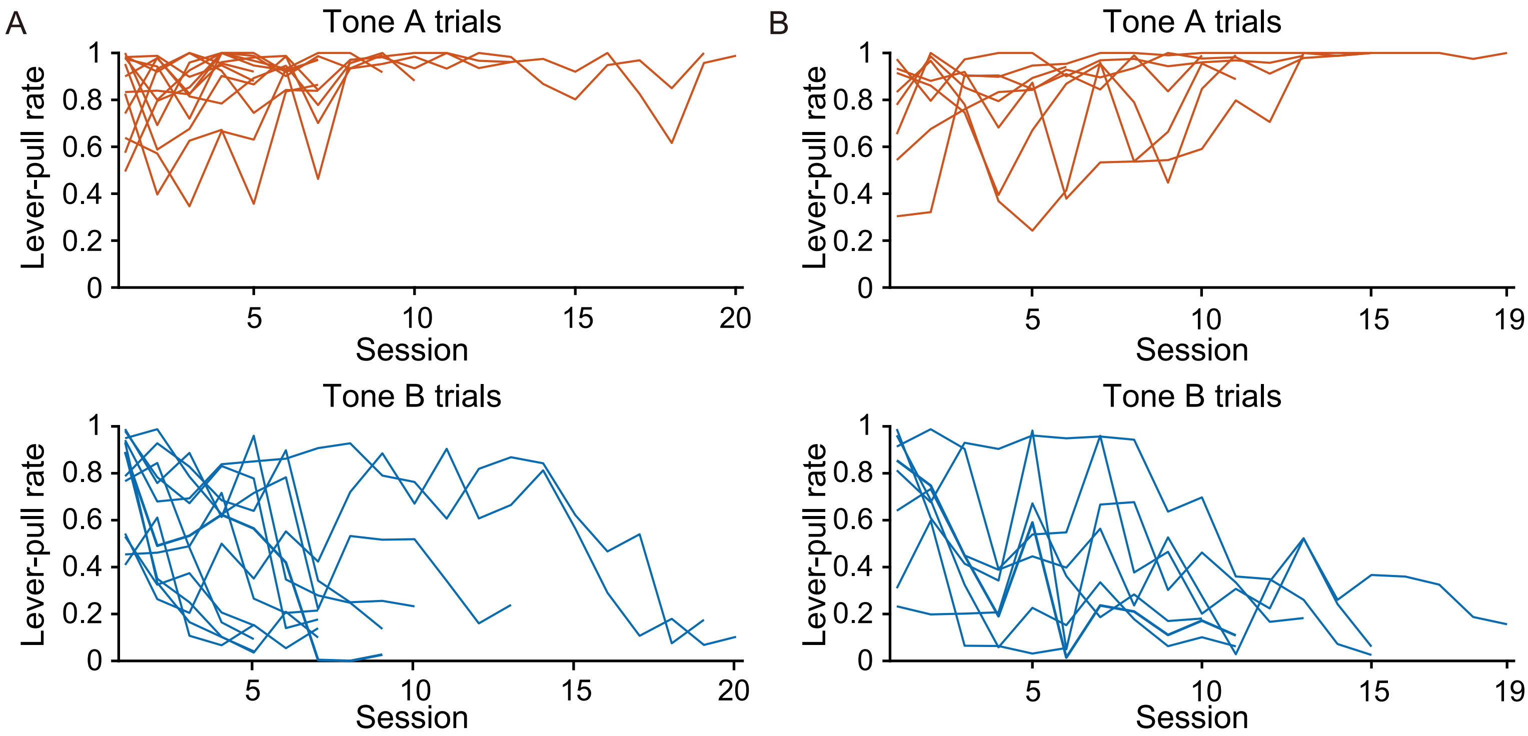

Supplementary Figure 1. Changes in lever-pull rate in all mice

(A) Individual changes in lever-pull rate in tone A (top) and B (bottom) trials across sessions in condition 1 (n = 13 mice).

(B) Individual changes in lever-pull rate in tone A (top) and B (bottom) trials across sessions in condition 2 (n = 8 mice).

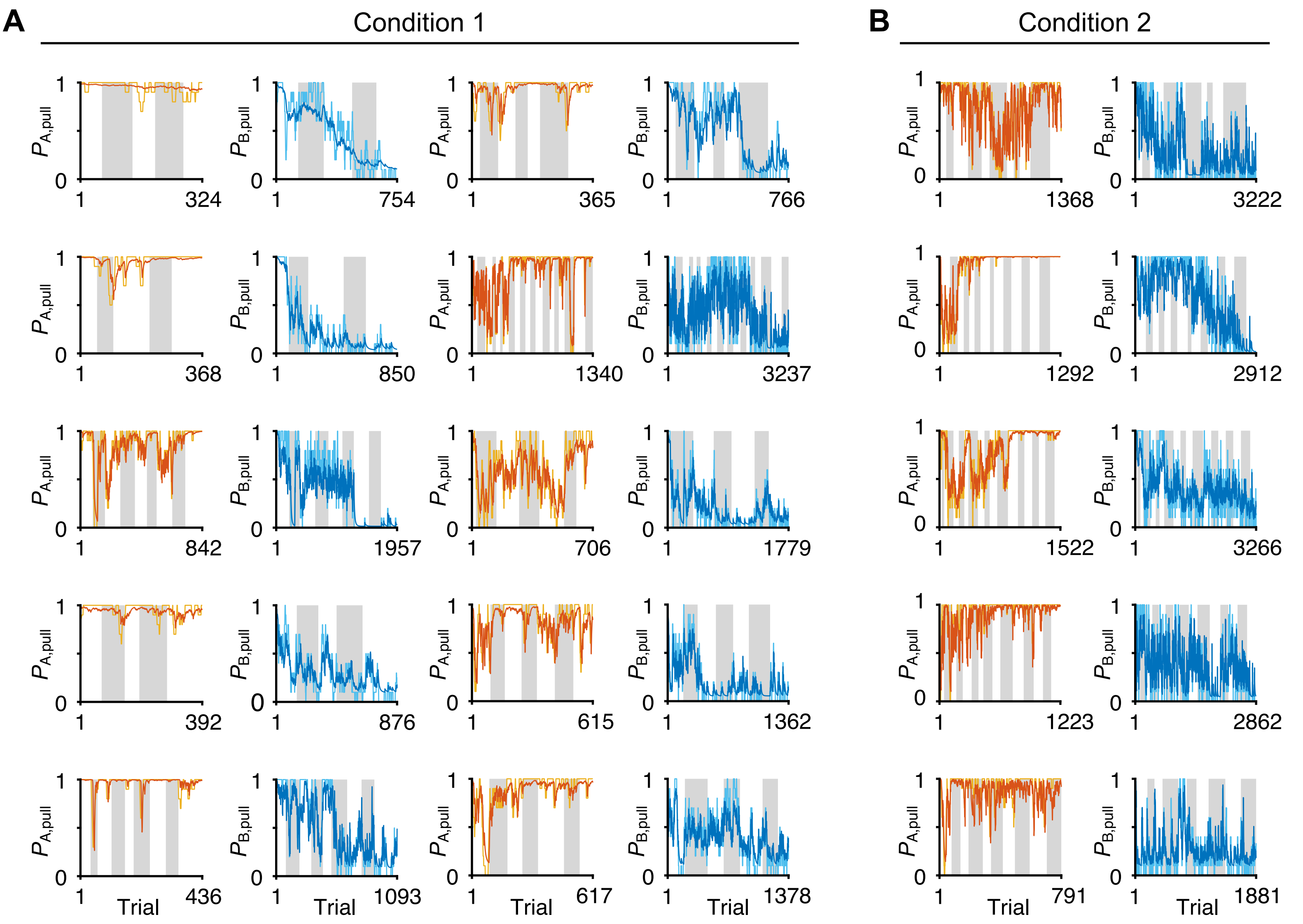

Supplementary Figure 2. Pull-choice probabilities predicted by the saving-F model in all mice except for those shown in Figure 4

(A, B) Time course of the 10-trial moving-average of the actual pull-choice (light colors) and predicted pull-choice probabilities (dark colors) across multiple concatenated sessions in condition 1 from ten mice (A) and condition 2 from five mice (B). None of these mice are shown in Figure 4. Pull-choice probabilities in tone A trials are in the leftmost and third from left subimages in (A), and in the left subimages in (B), whereas those in tone B trials are in the second left and rightmost subimages in (A), and in the right subimages in (B). Even sessions are shaded.

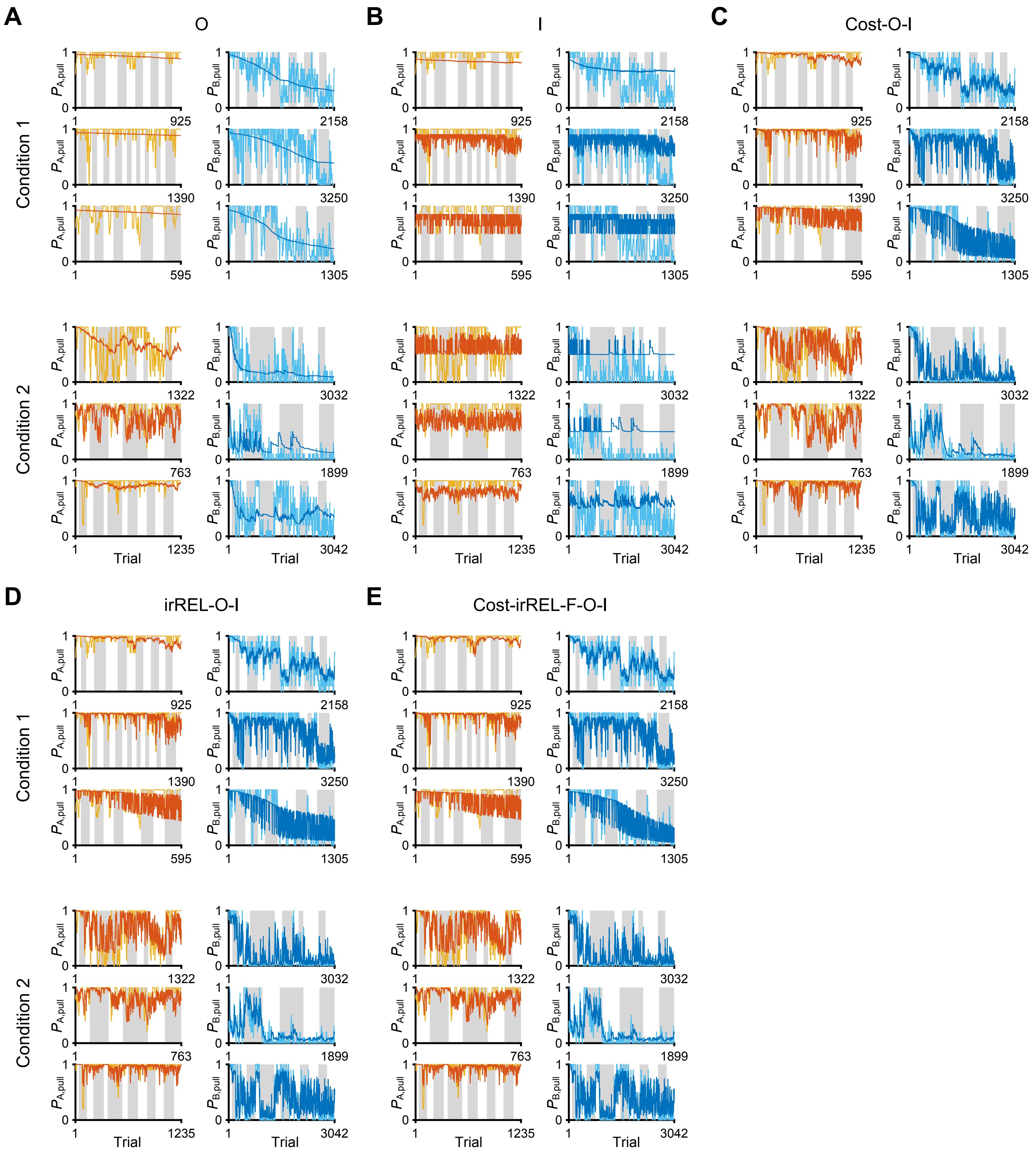

Supplementary Figure 3. Predictions by other models with the offset term and/or inertia of the choice

Six representative predicted pull-choice probabilities of the models in tone A (left) and B (right) trials in conditions 1 (top three rows) and 2 (bottom three rows). Orange and cyan traces represent the 10-trial moving-average of the actual pull-choice (the same as in Figure 3). Red and blue traces represent the predicted pull-choice probability in O (A), I (B), cost-O-I (C), irREL-O-I (D), and cost-iREL-F-O-I (E) models.

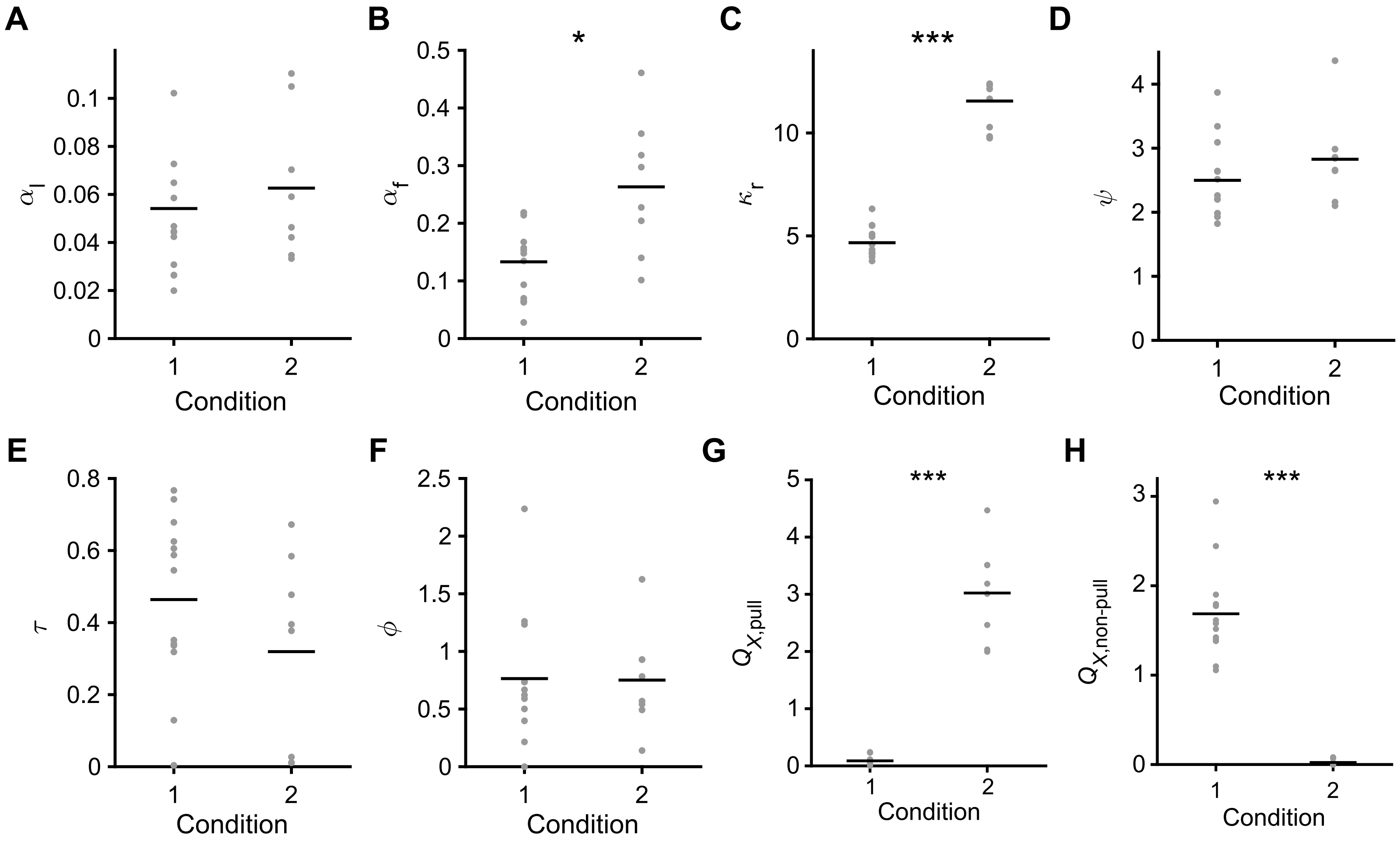

Supplementary Figure 4. Model parameters and Q-values in the saving-F-I model

(A) Learning rate (α_l), mean ± standard deviation, 0.054 ± 0.030 in condition 1, 0.062 ± 0.030 in condition 2, p = 0.491.

(B) Forgetting rate (α_f), 0.133 ± 0.064 in condition 1, 0.263 ± 0.118 in condition 2, *p = 0.0153.

(C) Subjective goodness of water reward (κ_r), 4.660 ± 0.753 in condition 1, 11.55 ± 1.49 in condition 2, ***p = 1.92×10^{-4}.

(D) Subjective goodness of covert reward (ψ), 2.498 ± 0.614 in condition 1, 2.827 ± 0.699 in condition 2, p = 0.180.

(E) Time constant of choice history (τ), 0.464 ± 0.236 in condition 1, 0.319 ± 0.268 in condition 2, p = 0.328.

(F) Subjective tendency to repeat the same choice (φ), 0.764 ± 0.561 in condition 1, 0.751 ± 0.438 in condition 2, p = 0.914.

(G) Pull values in tone X trials in the last session, X is B in condition 1 and A in condition 2. ***p = 1.92×10^{-4}.

(H) Non-pull values in tone X trials in the last session, X is B in condition 1 and A in condition 2. ***p = 1.92×10^{-4}.

Gray dots represent individual mice, and black bars represent the mean.
